# Molecular Typing of Mastitis-Causing *Staphylococcus aureus* Isolated from Heifers and Cows

**DOI:** 10.3390/ijms14024326

**Published:** 2013-02-21

**Authors:** Lívia Castelani, Aline Franciele Silva Santos, Mariana dos Santos Miranda, Luiz Francisco Zafalon, Claudia Rodrigues Pozzi, Juliana Rodrigues Pozzi Arcaro

**Affiliations:** 1 Institute of Animal Science, Dairy Cattle Center, Nova Odessa 13460, São Paulo, Brazil; E-Mails: alinefran18@yahoo.com.br (A.F.S.S.); mariana@iz.sp.gov.br (M.S.M.); pozzi@iz.sp.gov.br (C.R.P.); 2 Embrapa Southeast Livestock, São Carlos 13560, São Paulo, Brazil; E-Mail: luiz.zafalon@embrapa.br

**Keywords:** epidemiology, heifers, PFGE

## Abstract

*Staphylococcus aureus* is among the main etiologic agents of bovine mastitis. A total of 83 isolates of *S. aureus* from mammary glands of primiparous heifers were collected in the prepartum, calving and during lactation. For lactating cows, a total of 27 isolates of *S. aureus* from mammary glands were collected during lactation. The samples were taken in two dairy farms located in Sao Paulo State, Brazil. The highest frequency of *S. aureus* isolation in heifers was at the end of lactation. Strains were typified through Pulsed-field gel electrophoresis (PFGE) and grouped according to patterns of restriction enzyme SmaI. PFGE generated seven clonal profiles that were grouped into three different lineages, with the LA lineage being predominant and identified in heifers, as well as in the cows from the two regions studied. It was concluded that the cows showed a significant source of dispersion of *S. aureus*. At the first lactation the heifers were infected by the same clonal profiles of *S. aureus* which were isolated from multiparous lactating cows. The heifers were infected during milking over the months of lactation.

## 1. Introduction

*Staphylococcus aureus* is one of the main pathogens isolated from contagious bovine mastitis cases in many countries. Although *S. aureus* is isolated less frequently from heifers than coagulase-negative staphylococci [1], the former continues to be an important pathogen due to its difficult control and high prevalence of antimicrobial resistance [2].

Mastitis in heifers poses a potential threat to milk production and udder health since development of the milk secretory tissue occurs mainly during first pregnancy, affecting future lactations [1]. Heifer mastitis has been extensively studied over the past decades [3–5], but it still remains unclear how heifers are infected with the etiological agents of mastitis. Infection may be caused by bacteria that are part of the normal teat skin flora and oral cavity of heifer calves that suck on each other, or by microorganisms present in the environment of these animals (bedding, manure) that are transmitted by flies. Another hypothesis is the transmission of these pathogens from older cows kept together with heifers [6].

Genotyping analysis of *S. aureus* isolated from bovine milk is an important tool in epidemiological studies of heifer mastitis that contributes to the understanding of pathogen dissemination. In addition, genotyping may help elaborate more effective control strategies and hygiene education programs throughout the production chain. Pulsed-field gel electrophoresis (PFGE) is the gold standard for the identification of bacterial lineages which presents high discriminatory power [7].

The objective of the present study was to determine the incidence of *S. aureus* isolated from primiparous heifers during prepartum, calving and lactation and to compare strains isolated from lactating cows by PFGE to better understand the mechanisms of dispersal of the pathogen.

## 2. Results

### 2.1. Isolation of *S. aureus*

**Figure 1:**
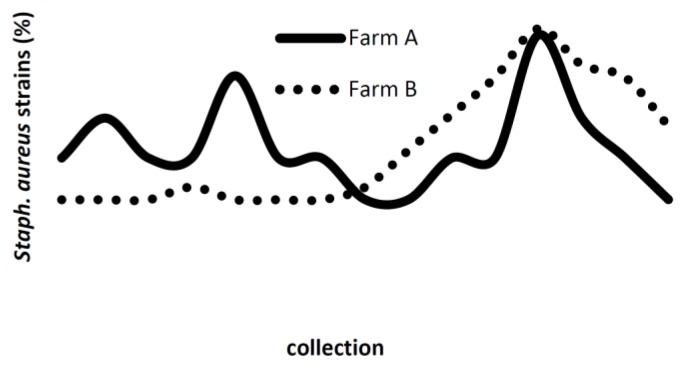
Absolute frequency of *S. aureus* isolated from mammary glands secretion from heifers during prepartum (1th), calving (2th), milk at 10 days postpartum (3th) and monthly during lactation (4th–15th).

A total of 110 isolated were identified as *S. aureus*. Among the 2596 mammary glands secretion samples collected from heifers, *S. aureus* were isolated from 83 (3.2%). Only one isolated was found during the prepartum period and two on the day of calving on farm A (Figure 1). Twenty-seven (10.5%) *S. aureus* were isolated from the 257 milk samples collected from lactating cows.

### 2.2. PFGE

**Figure 2:**
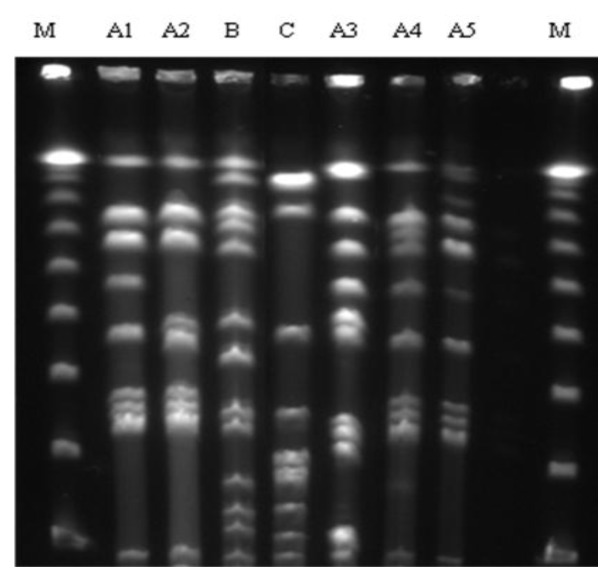
Pulsed-field gel electrophoresis (PFGE) profiles of *S. aureus* digested by SmaI isolated from mammary glands secretions of heifers and cows from farms A and B. M: Molecular marker-DNA-PFGE λ 50,000 bp; A1, A2, B, C, A3, A4, A5: PFGE profile.

In that study, 110 isolated were typified through the technique of PFGE. More than eight chromosomal DNA fragments were generated for each strain analyzed. Analysis of these fragments led to the identification of seven distinct clonal profiles grouped into three different lineages (Figure 2).

Only two different PFGE profiles were detected on farm A (A2 and B). In contrast, all PFGE profiles were identified on farm B, with a predominance of A1 profile in both heifers and cows (Table 1).

Comparison of the strains in terms of lineages between and within farms showed a predominance of LA lineage. In addition, A1 and A2 profiles (Figure 2) differed by only one band, a finding suggesting that the clones were originally identical at the two sites and are now evolving independently.

## 3. Discussion

The *S. aureus* frequency isolation from heifers samples were low (3.2%) throughout the experimental period. The prevalence of *S. aureus* in intramammary infections of heifers varies between studies and herds, but is generally lower than that of coagulase-negative staphylococci. Pankey and Co-workers isolated *S. aureus* from 2.6% of samples collected 3 days after calving from heifers on 11 dairy farms in Vermont [8]. Piepers and Co-workers detected this pathogen in 3.5% of postpartum samples [5].

Except for one heifer belonging to farm A from which *S. aureus* was isolated during prepartum and calving, the highest frequencies of isolation were observed during lactation (Figure 1). In addition, PFGE showed the same PFGE profiles in heifers and cows from both farms, except for the heifer mentioned above. The heifers were infected during milking over the months of lactation. Mechanical milking machines are important sources of staphylococci in dairy herds since they can be contaminated with microorganisms from the animal’s skin, milk, or hands of the operator [9]. In an epidemiological study of bovine mastitis, Smith and Co-workers demonstrated the importance of milking machines as a source of *S. aureus* [10]. The authors detected the two most prevalent clonal profiles isolated from bovine milk in samples collected from milking machine liners. The lack of good management practices during milking may contribute to the dissemination of pathogenic microorganisms. One strategy to control heifer mastitis is to milk them before cows.

Another important factor is to raise heifers in separate barns. Studies have shown an increased risk of intramammary infection in heifers raised together with older cows and that the same pathogens involved in the etiology of mastitis in cows were also present in heifers [1]. The mammary gland of heifers infected with coagulase-positive *Staphylococcus* represents a source for transmission to healthy animals of the herd [11].

The PFGE profile detected in only one heifer was B profile. This profile was isolated during the prepartum and postpartum period. Newly calved heifers infected with *S. aureus* may represent an important source of infection for healthy animals during milking [11]. This heifer carried a distinct PFGE profile when compared to the other animals of farm A and is therefore an important source for transmission of a different lineage on this farm. The detection of clonal profiles in a herd that differ from those commonly isolated may be the result of selection pressure and these profiles are specific for a certain geographic region [10]. An emerging lineage may be more pathogenic and transmitted easily within the herd, even under excellent milking conditions [12]. At the same time, clonal B profile was also isolated from only one sample collected from a cow belonging to farm B.

Although a large number of molecular profiles of *S. aureus* are involved in the etiology of bovine mastitis in the world, certain clones tend to predominate in different geographic regions [13,14]. In the present study, there was a predominance of the LA lineage corresponding to 89.1% of all isolates (Tables 1). Smith and Co-workers demonstrated that a single clonal group is responsible for most intramammary infections in cattle and that this group shows a wide geographic distribution [15]. Some *S. aureus* lineages possess a combination of genes that confer the ability to cause and disseminate infection, and a limited number of clones are responsible for cases of bovine mastitis on different farms [16].

Knowledge of the epidemiology of *S. aureus* obtained by molecular typing contributes to the development of strategies for the control and prevention of intramammary infections. Studies investigating virulence factors of the most prevalent *S. aureus* strains will permit the identification and production of specific antigens for vaccine development. In addition, the adoption of good practices of milking hygiene and qualified operators are essential for the control of mastitis. Another relevant approach to eliminate predominant strains is the culling or separation of animals with chronic mastitis. The predominance of these lineages might be a consequence of increased resistance of the pathogen to the host immune response. In this respect, Su and Co-workers observed that strains carrying more frequent profiles of the *coa* gene are more resistant to the action of neutrophils than those carrying less frequent profiles [17].

## 4. Materials and Methods

### 4.1. Collection of Biological Material

The experiment was conducted on two experimental dairy farms in the State of Sao Paulo, Brazil, from March 2009 to February 2011. Mammary glands secretion samples were collected from 54 heifers at 60 days before calving; colostrum was collected on the day of calving and milk samples were collected at 10 days postpartum and monthly until the end of lactation. Milk samples were also collected from 68 lactating cows in a single sampling. Thirty-six Black-and-White Holstein heifers and 48 cows belonged to farm A, and 18 crossbred heifers and 20 cows belonged to farm B. Milk samples were collected from cows of farm A on September 2009 and from cows of farm B on December 2010. The samples were collected according to the guidelines of the National Mastitis Council [18].

### 4.2. Isolation and Identification

*S. aureus* were isolated by culture on 5% defibrinated sheep blood agar and identified by standard biochemical tests catalase test, coagulase production on rabbit plasma, mannitol fermentation, acetoin production, maltose and trehalose utilization [19].

### 4.3. Species Confirmation by PCR

The confirmation of *S. aureus* species was done by Polymerase Chain Reaction (PCR), according to the protocol and primers described by Martineau and Co-workers [20]. Bacterial DNA was extracted using the RTP^®^ Bacteria DNA Mini kit (Invitek, Berling, Germany) according to manufacturer instructions.

### 4.4. PFGE

Bacterial DNA for molecular typing by PFGE was prepared as described by McDougal and Co-workers [21]. PFGE was carried out using the CHEF-DR III system (Bio-Rad, Melville, NY, USA) and a 50,000-bp λ DNA PFGE molecular weight marker (Sigma-Aldrich, St. Louis, MO, USA). The DNA fragments were stained by immersion of the gels in 100 μL 0.5% TBE buffer containing 7.5% GelRed for 20 min. The gels were photographed using the GelDoc^®^ (Bio-Rad, Hercules, CA, USA) photodocumentation system for visualization of the different profiles.

### 4.5. Statistical Analysis

The PFGE results were analyzed and interpreted as described by Tenover and Co-workers [22]. Isolates were considered genetically indistinguishable (PFGE profile) when their restriction patterns were the same numbers of corresponding bands and the bands had the same apparent size. Isolates were considered closely related when their PFGE patterns differed in two or three bands. An isolate was considered the same lineage when their PFGE patterns differed from 4–6 bands. An isolate was considered unrelated when differ between seven or more bands.

The degree of homology between the typed strains was determined by the Dice coefficient [23] and cluster correlation coefficients were calculated by the Unweighted Pair Group Method with Arithmetic Mean (UPGMA) method. The restriction profiles were classified by hierarchical cluster analysis [24] using the Jaccard coefficient and Ward’s method [25] as measures of similarity between profiles. Statistical analysis was performed in the R environment, version 2.11.0 [26], using the Vegan package and base distribution packages.

## 5. Conclusions

The study concluded that cows represent an important source of dispersion of *S. aureus* to heifers. Raising heifers in separated lots and the implementation of a milking line system where cows are firstly milked are strategies for intra mammary infections prevention. Good milking practices also help to control mastitis once milking equipment also becomes a source of pathogen agents.

## Figures and Tables

**Table 1:** Absolute (AF) and relative frequency (RF) of *S. aureus* isolates from mammary glands secretion of heifers and cows

PFGE lineage	PFGE profile	Farm A				Farm B				Total	

		Heifers		Cows		Heifers		Cows			

		AF	RF	AF	RF	AF	RF	AF	RF	AF	RF
LA	A1	-	-	-	-	57	89.1	16	88.9	73	66.4
	A2	9	47.4	9	100.0	4	6.3	-	-	22	20.0
	A3	-	-	-	-	1	1.6	-	-	1	0.9
	A4	-	-	-	-	-	-	1	5.6	1	0.9
	A5	-	-	-	-	1	1.6	-	-	1	0.9
LB	B1	10	52.6	-	-	-	-	1	5.6	11	10.0
LC	C1	-	-	-	-	1	1.6	-	-	1	0.9
	Total	19	100	9	100	64	100	18	100	110	100
